# The post-activation performance enhancement of sub-maximal load back squat with varied range of motion and intra-set fatigue on jump height in trained males

**DOI:** 10.1371/journal.pone.0356542

**Published:** 2026-08-19

**Authors:** Pengfei Xu, Liang Zhao, Changyu Tan, Duanying Li, Lin Xie, Wei Han

**Affiliations:** 1 Digital Physical Training Laboratory, Shandong Sport University, Rizhao, Shandong, China; 2 Graduate School, Shandong Sport University, Jinan, Shandong, China; 3 School of Competitive Sports, Shandong Sport University, Rizhao, Shandong, China; 4 Shandong Swimming Management Center, Shandong Sport Training Center, Jinan, Shandong, China; 5 School of Athletic Training, Guangzhou Sport University, Guangzhou, Guangdong, China; 6 Faculty of Health Sciences and Sports, Macao Polytechnic University, MacaoChina; Burdur Mehmet Akif Ersoy Üniversitesi: Burdur Mehmet Akif Ersoy Universitesi, TÜRKIYE

## Abstract

**Background:**

Post-activation performance enhancement (PAPE) is a strategy in sports science that improves athletic performance through preconditioning muscle activity. This study investigates the acute effects of sub-maximal load back squats with different ranges of motion (ROM) and intra-set fatigue on countermovement jump (CMJ) height.

**Methods:**

This study employed a counterbalanced, repeated-measures experimental design to investigate the acute effects of sub-maximal load back squat with varied ROM and intra-set fatigue on CMJ height in trained males. A total of 12 resistance-trained athletes participated in a single-set protocol intervention comprising 4 conditioning activities (CAs): (i) deep squats with proximity-to-failure (SDP); (ii) deep squats to failure (SDF); (iii) half squats with proximity-to-failure (SHP); and (iv) half squats to failure (SHF). CMJ height was measured before the CAs and at 4, 8, and 12 minutes post-intervention, while mean velocity was continuously monitored during the CAs.

**Results:**

No significant differences were observed in initial mean velocity across conditioning activities (F = 1.10, η_p_² = 0.07, *p* = 0.36). Final repetition mean velocity differed significantly across conditions (F = 6.67, η_p_² = 0.31, *p* < 0.01), with lower velocities and a greater number of repetitions observed in the failure conditions compared with the proximity-to-failure conditions (*p* < 0.01). A significant condition × time interaction was found for CMJ height (F = 2.231, η_p_² = 0.13, *p* = 0.02). CMJ height increased at 4 minutes post-CA in the SDP and SHP conditions (*p* < 0.01 and *p* = 0.03, respectively), while increases occurred at 8 minutes post-CA in the SDF and SHF conditions (*p* = 0.03 and *p* < 0.01, respectively). A significant between-group effect was also observed (F = 3.949, η_p_² = 0.21, *p* = 0.01). No between-group differences were present at 4 minutes post-CA (*p* = 0.24), but significant differences emerged at 8 and 12 minutes post-CA (both *p* < 0.01).

**Conclusion:**

Both proximity-to-failure and repetitions-to-failure protocols performed at 87% 1RM effectively enhanced CMJ performance. However, peak performance occurred earlier following the proximity-to-failure protocols, and half squats elicited greater enhancement effects than deep squats. These findings suggest that 3 repetitions of the half squat at 87% 1RM may represent a time-efficient strategy for inducing PAPE when explosive performance is required shortly before competition or training.

## 1. Introduction

In the field of sports, athletes must effectively utilize explosive power, which is essential for optimizing performance and achieving competitive success [1,2]. Coaches and sports scientists have long sought methods and strategies to effectively enhance athletes’ explosive power [3]. Among these approaches, post-activation performance enhancement (PAPE) has received considerable attention and is defined as the improvement in athletic performance following voluntary muscle activation [4]. The enhancement typically emerges after several minutes and may persist for a period of time [5]. Although PAPE may be influenced by several physiological factors, including increases in muscle temperature, muscle hydration, and neuromuscular activation, it is primarily considered a performance-based phenomenon independent of the underlying PAP-related mechanisms [4,6]. Previous studies have demonstrated that specific conditioning activities (CAs), such as back squats, can induce PAPE and improve performance in explosive movements such as the countermovement jump (CMJ) [7–9]. Back squats are a fundamental resistance exercise used to develop lower-body maximal strength and neuromuscular capacity, both of which are strongly associated with improvements in jumping, sprinting, and other sport-specific athletic tasks. Therefore, back squats are frequently incorporated into conditioning and training strategies aimed at enhancing explosive performance. In addition, they are also widely used due to their practicality, requiring minimal space, equipment, and financial resources [10,11].

Countermovement jump height is a key indicator for evaluating muscular strength and overall athletic performance [12–15]. It is widely used across various sports for performance monitoring and has been further developed as a distinct athletic skill [12,16]. Research has demonstrated that weighted back squats can effectively induce PAPE, thereby enhancing CMJ performance and explosive power-related outcomes [17]. However, the efficacy of PAPE induced by back squats on CMJ performance depends on multiple factors, such as training experience, gender, strength level, load intensity (%1RM), contraction type, ranges of motion (ROM), fatigue level (e.g., number of repetitions, velocity loss (VL)), training volume (sets), intra-contrast rest period (ICRP), and the type of subsequent activity [1,7,18–20]. Studies have shown that moderate loads (~65% 1RM) can acutely induce PAPE and improve CMJ performance, whereas submaximal loads (85–90% 1RM) often lead to greater performance enhancements [5]. Furthermore, evidence suggests that for experienced athletes with a back squat relative strength of approximately 1.5–2 times body mass, the optimal load for enhancing CMJ height when using the squat as a CA is around 5RM, corresponding to roughly 87% 1RM [21–24].

Fatigue is a key factor influencing the manifestation of PAPE [4,5,25]. In current research, intra-set fatigue, defined as the level of fatigue experienced during each training set, is influenced by the proximity to muscular failure and is often studied as a variable impacting PAPE [26]. Repetitions to failure refers to the continuous completion of repetitions, until the athlete is unable to complete the concentric phase of an exercise [27]. Conversely, proximity to failure involves ending the set with repetitions in reserve to avoid complete neuromuscular exhaustion [28]. Long-term training performed with repetitions-to-failure protocols does not necessarily lead to superior improvements in maximal strength and muscle hypertrophy compared with proximity-to-failure protocols (i.e., terminating a set before reaching volitional failure), and may even result in significantly greater systemic fatigue [29,30]. However, when repetitions-to-failure protocols are employed as CAs, they can elicit CMJ height improvements comparable to those observed following proximity-to-failure protocols. [21,31–35]. Although repetitions-to-failure protocols progressively increase motor unit firing rates and muscle fiber activation as repetitions accumulate [29,30,36], the greater fatigue associated with these protocols may partially offset the benefits of enhanced neuromuscular activation. In contrast, proximity-to-failure protocols with submaximal loads produces less fatigue and allows quicker recovery to express performance enhancement effects [37]. Consequently, despite the different physiological demands imposed by these approaches, both repetitions-to-failure and proximity-to-failure protocols appear capable of enhancing CMJ height. Nevertheless, the magnitude and timing of the enhancement effect reported across studies appear to vary, regardless of load intensity or proximity to failure, highlighting the need for direct comparative investigations.

Range of motion is another critical factor influencing PAPE [7,38]. Variations in squat ROM significantly affect the extensor muscles and the magnitudes of the associated joint moments [39,40]. As ROM increases, the hip and knee joints exhibit greater flexion moments, and a sticking region typically emerges during deeper squats [22,41]. In PAPE-related research, Esformes and Bampouras [1] reported that both quarter squats and parallel squats elicited PAPE; however, the parallel squat produced a greater enhancement effect. Similarly, Krzysztofik et al. [18] reported that, under the same loading intensity, the standard-range bench press elicited a greater enhancement effect than bench press performed with either a larger or smaller ROM. These findings indicate that variations in ROM influence the magnitude of the enhancement effect, supporting the observations of Esformes and Bampouras that ROM is an important determinant of PAPE. In previous research, both repetitions to failure and proximity to failure interventions were shown to significantly improve CMJ height, but the squat depth requirements varied across studies. For instance, in studies by Kilduff et al. [21,31] on proximity to failure interventions, participants were instructed to squat until the hip crease was below the patella. In contrast, Mitchell et al. [33] and Sireiro et al. [35] required participants in repetitions to failure interventions to squat until the knee angle reached 90°. Therefore, without controlling for ROM, comparing the enhancement effects of repetitions to failure and proximity to failure is not scientifically rigorous.

Despite extensive research on the use of submaximal-load back squats to enhance jump performance in trained male athletes, several key issues remain unresolved. Firstly, under squat conditions involving repetitions to failure and proximity to failure, the PAPE effects on CMJ height improvement vary across studies, and direct comparisons between the two are limited. Secondly, related research lacks consistency in the ROM employed during squat interventions, and no studies have directly compared the acute effects of deep squats and half squats on jump height. Therefore, this study aims to determine the effectiveness of intra-set fatigue (repetitions to failure vs proximity to failure) on enhancing CMJ height in male athletes under deep squat conditions (thighs in contact with calves) and half squat conditions (thighs and calves at a 90° angle). Although deeper squats have been shown to induce greater PAPE in highly trained athletes with high relative strength (≥2.0), who are capable of rapid recovery from the associated fatigue [1,21], individuals with moderate relative strength (~1.5) may derive greater benefit from half squats [35]. The reduced ROM in the half squat decreases total mechanical work and fatigue, which may better align with the recovery capacity of this population within a given time window. Given that the participants in the present study exhibit similar strength levels to those reported by Sirieiro et al. [35], We hypothesized that all CAs will lead to improvements in CMJ height in moderately strong males, with half squats producing greater enhancement effects than deep squats. Additionally, we anticipate that the effects of repetitions to failure and proximity to failure on CMJ height will be similar. The findings of this study will provide practitioners with practical guidance for selecting appropriate CAs to enhance explosive performance in training and competitive settings.

## 2. Materials and methods

### 2.1 Participants

A sample size estimation was conducted using G*Power software (version 3.1.9.7, Dusseldorf, Germany) with the parameters “ANOVA, repeated measures, within factors”. The analysis indicated that detecting an effect size of *f* = 0.38 would require a minimum of 11 participants completing 4 repeated measurements (α = 0.05, power = 0.80, correlation among repeated measures = 0.50) [42]. To account for potential dropouts, 12 male athletes were recruited to participate in the study (Table 1). These participants were amateur athletes with regular resistance training backgrounds. The study participants had a relative squat strength of 1.5–2 times their body mass and at least 2 years of resistance training experience prior to inclusion in the study. The male athletes were allowed to withdraw from the experiment at any time.

**Table 1:** Descriptive characteristics of the study participants.

Variables	Mean ± SD
Age (years)	20.2 ± 0.9
Height (m)	1.77 ± 0.05
Body Mass (kg)	77.5 ± 10.5
Body Mass Index (kg·m^-^²)	24.7 ± 3.3
Experience in resistance training (years)	3.5 ± 1.3
Relative 1RM deep squat (kg·kg^-1^)	1.7 ± 0.1
Relative 1RM half squat (kg·kg^-1^)	1.9 ± 0.2

Mean: mean values; SD: standard deviation; RM: repetition maximum; Relative 1RM: ratio of 1RM to body mass.

The inclusion criteria were: (i) free from neuromuscular and musculoskeletal diseases, (ii) resistance-trained, and (iii) self-reported satisfactory health status. Participants were excluded based on self-reported information if they (i) engaged in strenuous activity within 24 hours before the experiment, or (ii) consumed alcohol, caffeine, or other stimulant drinks within 4 hours before the experiment. Participants were recruited between 01/09/2022 and 31/10/2022. All participants were informed about the purpose, procedures, and potential risks and benefits of the study. Written informed consent was obtained from all participants prior to their participation. The study protocol was approved by the Sports Science Ethics Commission at the Shandong Sport University, China (2021039), and performed according to the ethical standards of the Declaration of Helsinki, 2013.

### 2.2 Experimental procedures

**Fig 1:**
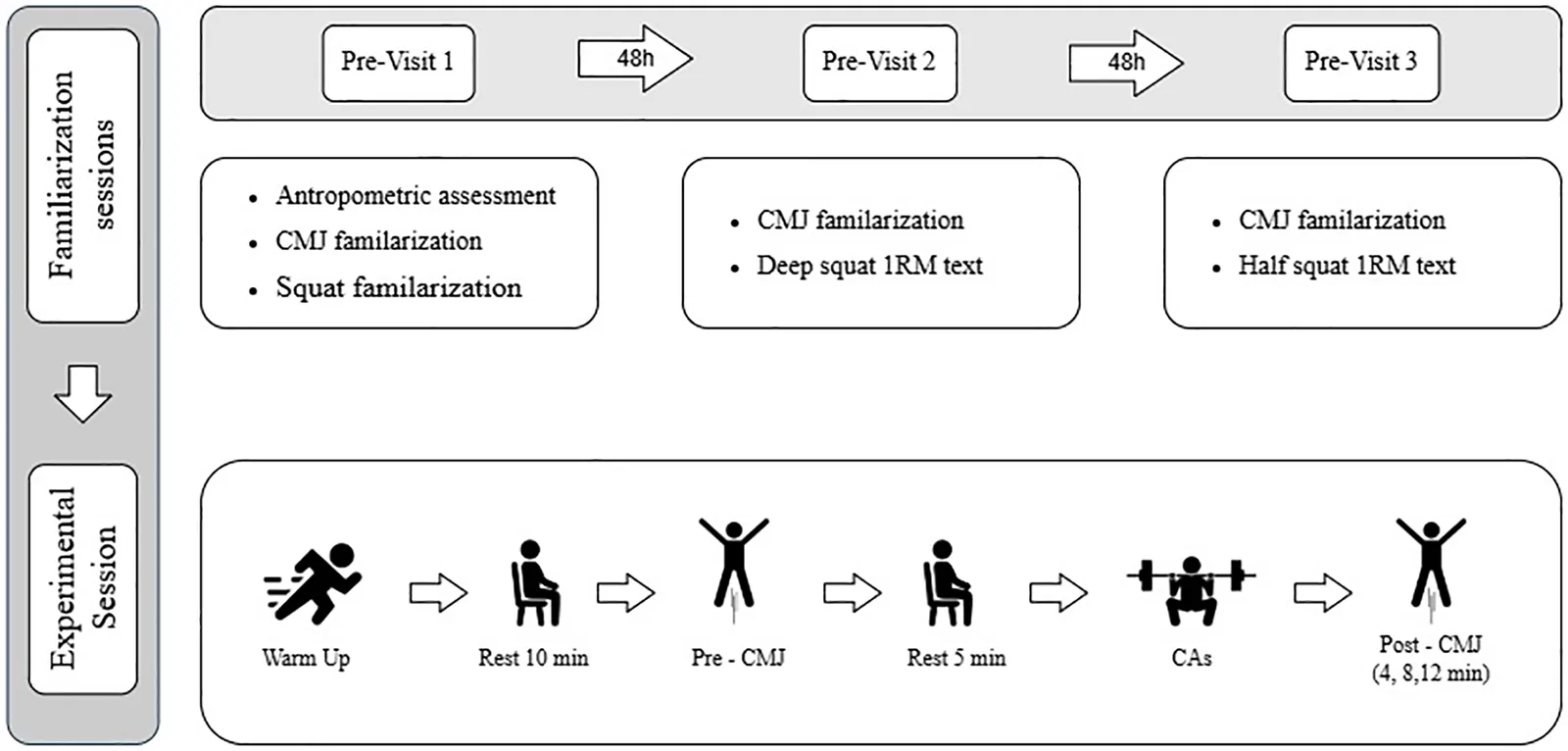
Schematic representation of the study design. RM: repetition maximum; CMJ: countermovement jump; CA: conditioning activity.

To investigate the effects of ROM and intra-set fatigue on PAPE, participants attended 3 familiarization sessions and 4 experimental sessions over a period of three weeks, with each session separated by at least 48 hours. The familiarization sessions involved anthropometric measurements and determination of the one-repetition maximum (1RM) load for both the deep squat and half squat intervention. Participants then performed one of the following conditions at 87% 1RM in a randomized order. The order of the 4 experimental conditions was determined using a card selection method: (i) deep squat with proximity-to-failure (SDP), (ii) deep squats to failure (SDF); (iii) half squats with proximity-to-failure (SHP); and (iv) half squats to failure (SHF). Under the proximity-to-failure protocols, participants performed 3 repetitions at 87% of 1RM, whereas under the repetitions-to-failure protocols, repetitions were continued until momentary failure. To investigate the acute effects of the squat exercise on subsequent CMJ height, a single set of 2 CMJs was performed 5 minutes before each CA and after 4-, 8-, and 12-min ICRPs following each CA (Fig 1) [43]. The mean CMJ height of the 2 jumps was used for statistical analysis.

#### 2.2.1 Familiarization session.

**Fig 2:**
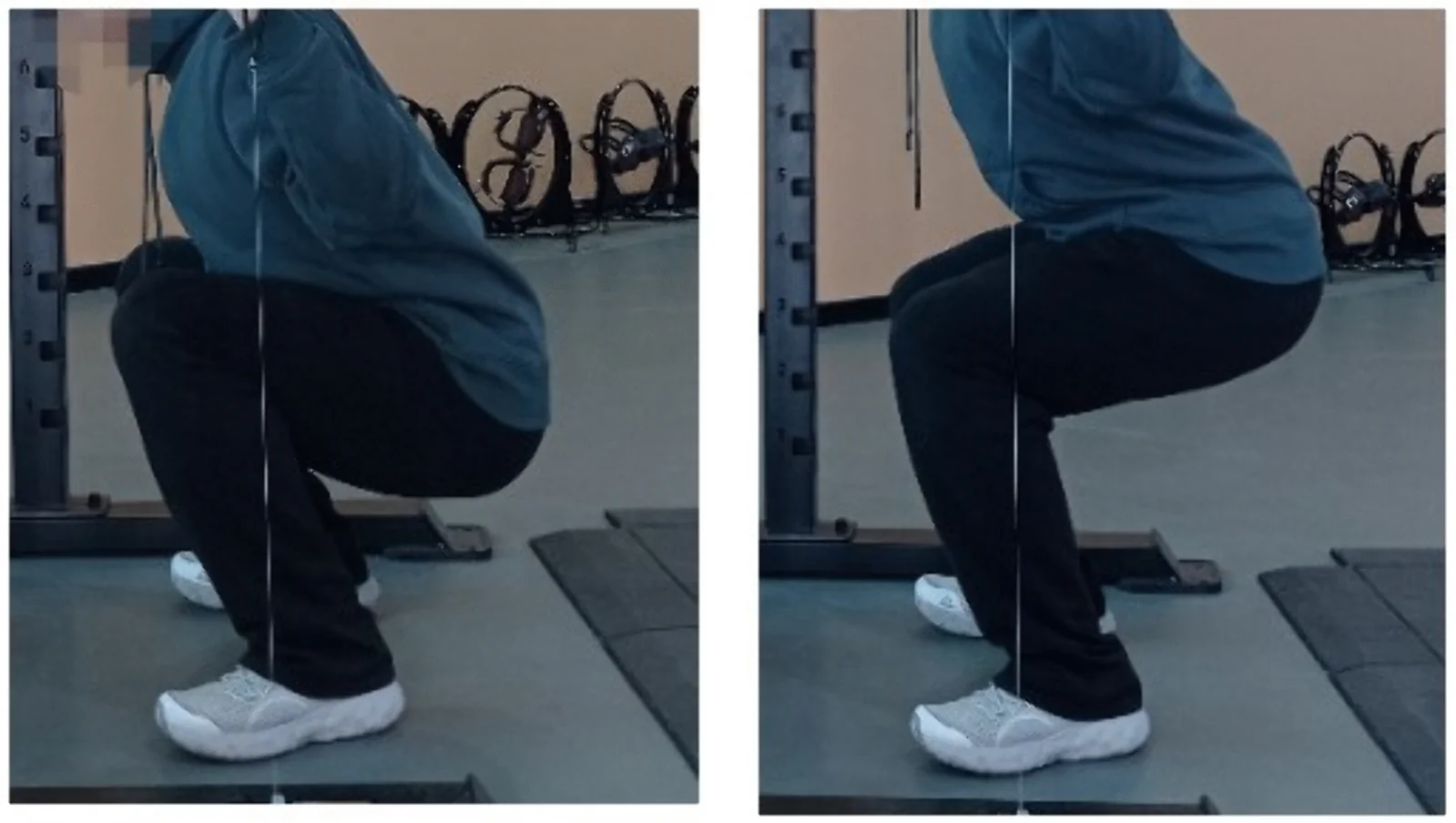
Deep squat and half squat techniques performed. Deep squat (Left), Half squat (Right).

The participants arrived in the laboratory at the same time of day as the upcoming experimental sessions (between 19:00 and 21:00). Initially, anthropometric measurements were taken, including height, body mass, and body mass index (GAIA KIKO, Jawon Medical Co., South Korea). Researchers assessed the participants’ performance techniques, providing instructions to ensure proper exercise execution. A deep squat was considered successful when the participant lowered themselves until the backs of their thighs touched their calves, while a half squat involved bending the knees to approximately 90° (Fig 2). Familiarization movements were performed with light loads, and participants repeated the exercises until they felt confident [22,44].

Participants completed 1RM testing for deep squat and half squat on 2 separate sessions, separated by at least 48 hours. The order of the 2 squat variations was counterbalanced across participants. Specifically, half of the participants (n = 6) performed the deep squat 1RM test first, followed by the half squat 1RM test, while the remaining participants (n = 6) performed the tests in the reverse order. According to the guidelines of the National Strength and Conditioning Association (NSCA), 1RM testing for the deep squat and half squat was performed using a barbell. Participants completed a 10-minute warm-up that included 5 minutes of jogging (7–8 km/h), followed by 5 minutes of general and specific warm-up exercises. The general warm-up involved dynamic stretching of the major lower-limb muscle groups (quadriceps, hamstrings, gluteal muscles, and calf musculature). The specific warm-up consisted of 5 unloaded squats performed at the designated depth and 3 CMJs. After a 5-minute rest interval, a progressive loading protocol was implemented for the 1RM assessment. Specifically, participants first performed a warm-up set consisting of 5–8 repetitions at approximately 40–60% of their estimated 1RM, followed by a second warm-up set of 3–5 repetitions at approximately 80% of their estimated 1RM based on prior training records and experience. The load was increased by 2.5–5 kg in each subsequent attempt until the participant could no longer perform the lift with proper technique. At least 3–5 min of rest was provided between maximal attempts. Participants were instructed to execute each repetition with a 2-second duration for the eccentric phase and maximal velocity during the concentric phase. Throughout the 1RM testing and subsequent CAs, trained observers were stationed on either side of the barbell to monitor lifting technique and ensure the safety of the participants [18].

#### 2.2.2 Experimental sessions.

After a warm-up similar to that conducted during the familiarization sessions, a 10-minute rest period was followed by the commencement of the baseline (BA) testing for CMJ. Five minutes after baseline testing, participants performed 1 of the 4 CAs, in a counterbalanced order. The 4 CAs were performed at 87% 1RM and consisted of: (i) deep squat with proximity-to-failure (SDP), (ii) deep squats to failure (SDF); (iii) half squats with proximity-to-failure (SHP); and (iv) half squats to failure (SHF) (Table 2). A set was terminated and considered to have reached repetition failure if the participant was unable to complete the concentric phase within 3 seconds or if a clear breakdown in lifting technique was observed. If the current repetition was not successfully completed, it was excluded from the analysis, and the corresponding value from the preceding successfully completed repetition was used. Following the completion of the CA, participants performed 2 CMJs after 4-, 8-, and 12-min ICRPs. Each CMJ testing consisted of 2 consecutive CMJ attempts, and the mean CMJ height of the 2 attempts was used for statistical analysis. Each condition was separated by a rest period of 48 hours. Participants were instructed to avoid high-intensity lower-body training throughout the duration of the study.

**Table 2:** Specifics of conditioning activities.

Condition	Range of motion	Set	Number of repetitions
SDP	Deep squat	1	3
SDF	Deep squat	1	Repeat until failure
SHP	Half squat	1	3
SHF	Half squat	1	Repeat until failure

SDP: deep squats with proximity-to-failure; SDF: deep squats to failure; SHP: half squats with proximity-to-failure; SHF: half squats to failure.

#### 2.2.3 Measurement of movement velocity during the conditioning activity.

A linear position transducer (GymAware Power Tool, Kinetic Performance Technologies, Australia) was used to measure the mean velocity during the CA. The velocity of the barbell was recorded at a sampling frequency of 50 Hz. Previous research has demonstrated good-to-excellent test–retest reliability of GymAware mean velocity measurements during back squat assessments performed at 40–90% of 1RM, with intraclass correlation coefficients (ICC) ≥0.75 and standard errors of measurement (SEM) of 0.03–0.05 m·s^-1^, supporting its suitability for monitoring resistance exercise velocity [45,46]. The external end of the cable was attached to the side of the barbell without providing any resistance. The device was positioned on the floor directly beneath the barbell, with its magnetic base placed on a weight plate to prevent any movement during each lift. VL thresholds were calculated using the following formula: [VL thresholds = (initial velocity – final velocity)/ initial velocity * 100%] [20]. In this calculation, the initial velocity refers to the barbell velocity recorded during the first repetition of each CA [47]. Final velocity represented the last successfully completed repetition prior to task failure.

#### 2.2.4 Measurement of counter-movement jump height.

CMJ height is widely used as an indicator of neuromuscular fatigue, with previous studies in athletic populations reporting good test-retest reliability (ICC = 0.82–0.88; coefficient of variation, CV = 3.9% − 4.8%) [48–50]. In addition, CMJ height measured using a linear position transducer has demonstrated excellent reliability (ICC = 0.96; CV = 6%) [16], supporting its use for repeated assessments when measurement procedures are standardized.

During the jump test, the linear position transducer was placed on the side of the participants and attached to a plastic dowel positioned on their shoulders. Participants started in a standing position, holding the bar with both hands. Upon hearing the command “go,” they were instructed to perform a quick downward movement to a self-selected depth, followed by a fast upward movement to jump as high as possible. After each jump, participants returned to the starting position, and the procedure was repeated for a total of 2 repetitions [16]. To observe the data in a standardized manner, we calculated the percentage change using the following formula: Δ% (percentage change) = (post – pre)/ pre * 100% [51].

### 2.3 Statistical analyses

All statistical analyses were conducted using SPSS software (version 25.0; SPSS, Inc., Chicago, IL) and were expressed as means with standard deviations (± SD). Additionally, the relative differences (percentages) between BA and post-CA values were calculated. Statistical significance was set at *p* < 0.05. The Shapiro-Wilk test and Mauchly’s test were employed to verify the normality and sphericity of sample data variances, respectively. A two-way (4 conditions × 4 time points) repeated measures analysis of variance (ANOVA) was used to compare CMJ height. When a significant main effect or interaction was observed, post hoc tests with Bonferroni correction were applied for pairwise comparisons. The range of mean differences was estimated using 95% confidence intervals. Effect sizes for the main and interaction effects were expressed as partial eta squared (η_p_²), with values of 0.01, 0.06, and 0.14 representing small, medium, and large effects, respectively [52,53]. Cohen’s *d* was calculated for post hoc pairwise comparisons and interpreted as trivial (< 0.20), small (0.20–0.50), medium (0.50–0.80), large (> 0.80) [54].

## 3. Results

In each CA, there were no significant differences in the initial movement velocity across the experimental conditions (F (3, 33) = 1.10, η_p_² = 0.07, *p* = 0.36), indicating that participants’ baseline states were similar across conditions. However, significant differences in final repetition mean velocity were observed between conditions for each CA (F (3, 33) = 6.67, η_p_² = 0.31, *p* < 0.01). Post hoc analyses revealed that the final repetition mean velocity in the repetitions-to-failure protocols was significantly lower than in the proximity-to-failure protocols. No significant differences were found in final repetition mean velocity between the proximity-to-failure protocols (*p* = 0.92) or between the repetitions-to-failure protocols (*p* = 0.66). Additionally, significant differences were observed in the number of repetitions completed across the different CAs (F (3, 33) = 40.15,η_p_² = 0.73, *p* < 0.01). The proximity-to-failure protocols involved an average of 3 repetitions, while the repetitions-to-failure protocols ranged from 7 to 9 repetitions, significantly more than the proximity-to-failure protocols. Table 3 presents the number of repetitions, along with the initial and final repetition mean velocities and velocity loss thresholds for each CA.

**Table 3:** Characteristics of the conditioning activities in the set.

Condition	Number of Repetitions	Initial velocity (m·s^-1^)	Final velocity (m·s^-1^)	VL thresholds (%)
SDP	3	0.5 ± 0.1	0.5 ± 0.1	2.8 ± 10.1
SDF	7.0 ± 2.4^#^	0.6 ± 0.1	0.4 ± 0.1^*^	24.0 ± 15.9
SHP	3	0.6 ± 0.1	0.5 ± 0.1	13.2 ± 13.7
SHF	9.3 ± 2.4^#^	0.6 ± 0.1	0.4 ± 0.1^*^	27.2 ± 7.6

SDP: deep squats proximity-to-failure; SDF: deep squats to failure; SHP: half squats proximity-to-failure; SHF: half squats to failure; VL: velocity loss; *: Significantly different from Initial velocity (p < 0.05); #: Significantly different from proximity-to-failure condition (p < 0.05).

**Fig 3:**
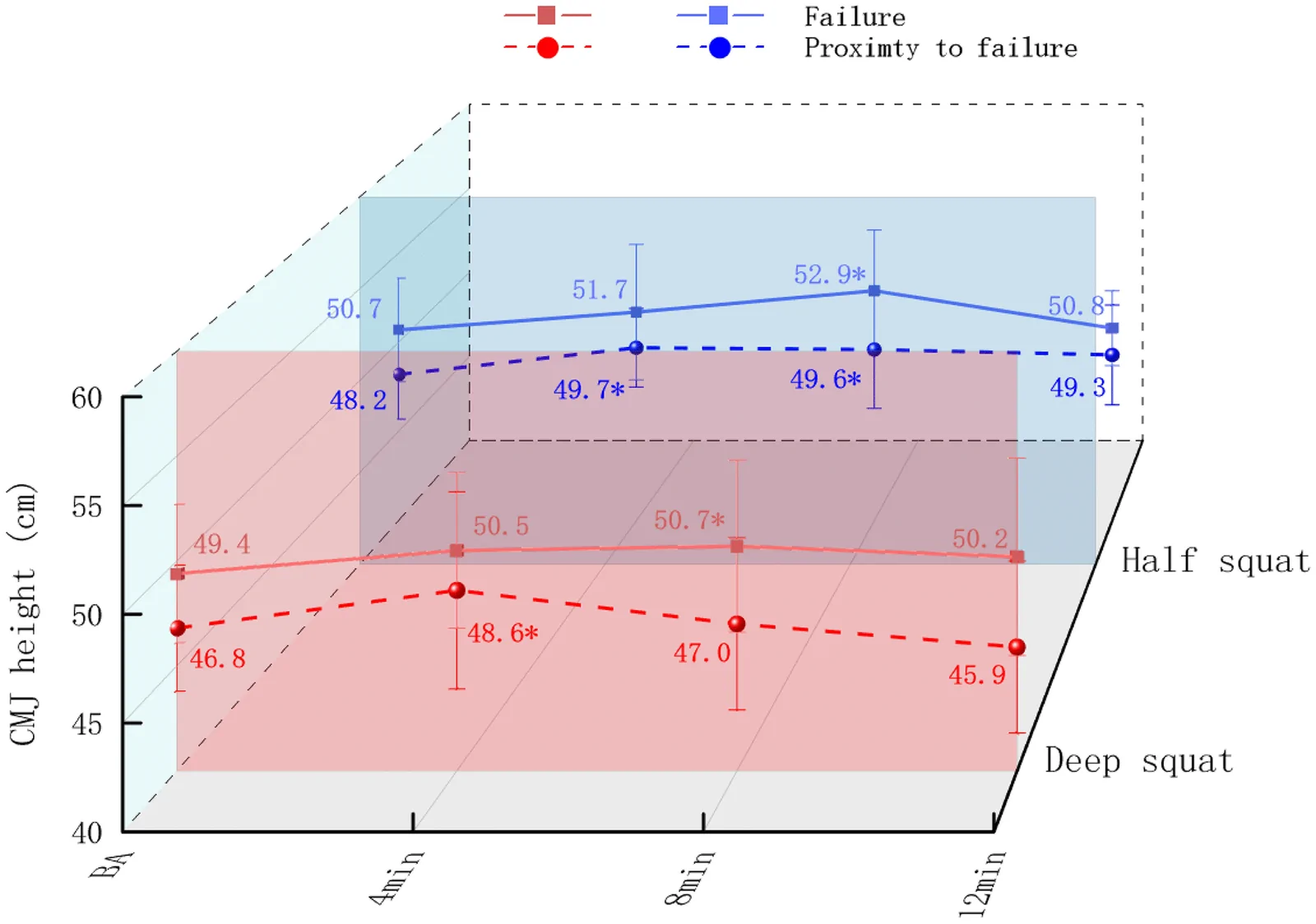
The impact of intra-set fatigue on CMJ Height. * Compared with baseline *p* < 0.05; BA: baseline.

**Fig 4:**
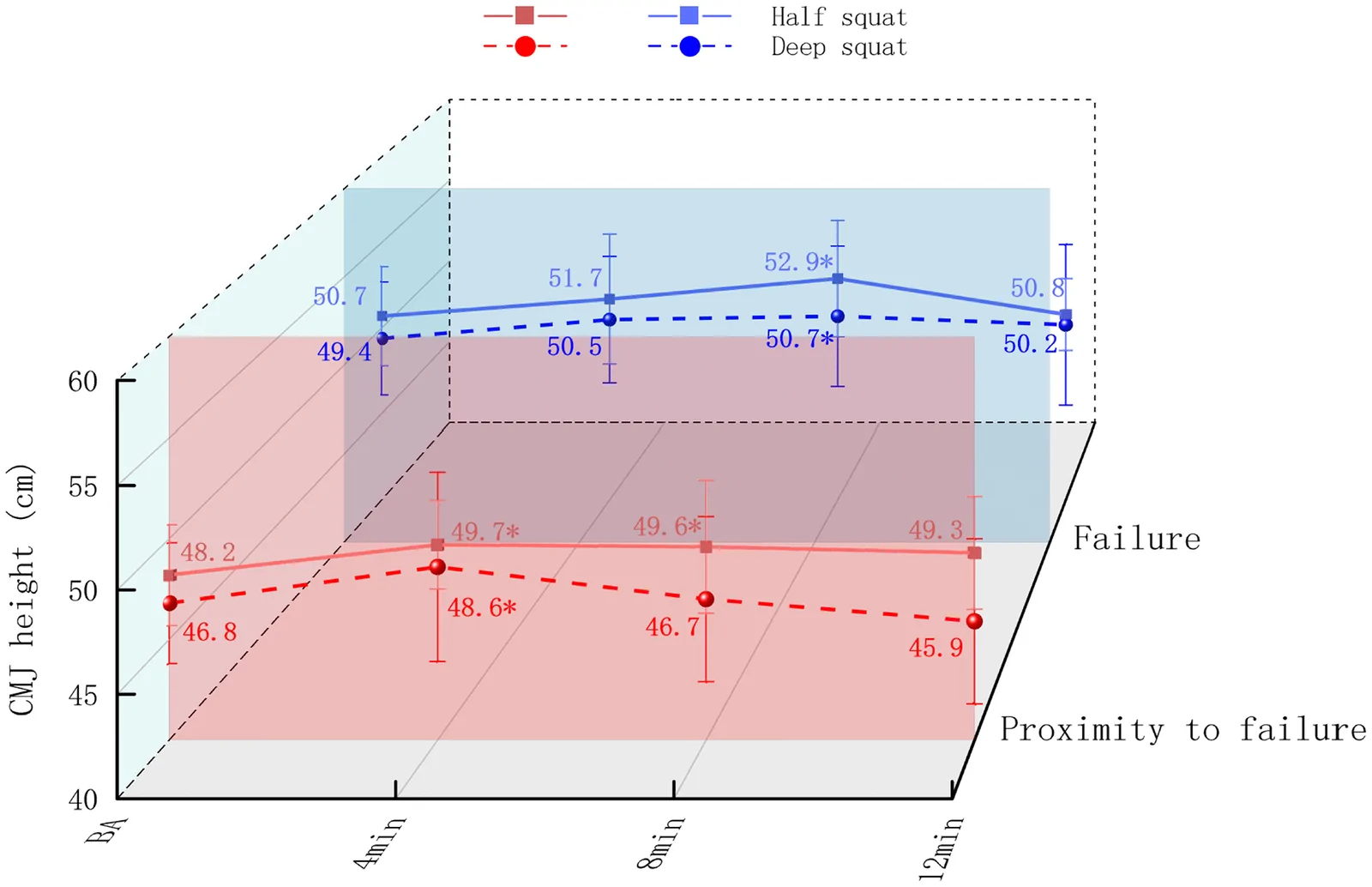
The impact of range of motion on CMJ Height. * Compared with baseline *p* < 0.05; BA: baseline.

A two-way repeated measures ANOVA showed a condition × time interaction for CMJ height (F (9, 99) = 2.23, η_p_² = 0.13, *p* = 0.02) and a main effect of time (F (3, 33) = 9.77, η_p_² = 0.18, *p* < 0.01). Post hoc comparisons indicated that CMJ height was significantly higher following all CA interventions compared with baseline. The greatest improvements were observed 4 min after the CA in the SDP and SHP conditions, whereas peak CMJ performance in the SDF and SHF conditions occurred at 8 min post-CA. A main effect of condition was also observed (F (3, 33) = 3.95, η_p_² = 0.21, *p* = 0.01). No significant differences among conditions were observed at 4 min post-CA; however, condition differences emerged at 8 and 12 min post-CA. Table 4 presents CMJ height values before and after each CA intervention, as well as the corresponding magnitude of improvement, with p-values and effect sizes reported for all pairwise comparisons. Figs 3 and 4 further illustrate the different temporal responses associated with intra-set fatigue and the similar temporal profiles observed between ranges of motion when fatigue level was matched.

**Table 4:** Comparison of pre- and post-CA counter-movement jump height (cm).

CA	Time Point	CMJ height (95%CI)	*p*	Δ%	*d* (95%CI)
SDP	BA	46.8 ± 3.0 (44.9 to 48.8)	——	——	——
	4min	48.6 ± 4.7 (45.6 to 51.6) *	0.01	3.7 ± 6.0	0.46 (−0.35 to 1.27)
	8min	47.0 ± 4.1 (44.4 to 49.6)	0.78	2.9 ± 4.4	0.06 (−0.75 to 0.85)
	12min	45.9 ± 4.1 (43.3 to 48.5)	0.14	−2.0 ± 4.6	0.25 (−1.06 to 0.55)
SDF	BA	49.4 ± 3.3 (47.3 to 51.5)	——	——	——
	4min	50.5 ± 3.7 (48.1 to 52.9)	0.09	2.2 ± 4.0	0.31 (−0.50 to 1.11)
	8min	50.7 ± 4.1 (48.1 to 53.4) *	0.03	2.7 ± 4.7	0.35 (−0.45 to 1.16)
	12min	50.2 ± 4.7 (47.3 to 53.2)	0.18	1.6 ± 4.6	0.20 (−0.60 to 1.01)
SHP	BA	48.2 ± 2.5 (46.7 to 49.8)	——	——	——
	4min	49.7 ± 2.2 (48.3 to 51.1) *	0.03	3.0 ± 3.7	0.64 (−0.22 to 1.42)
	8min	49.6 ± 3.3 (47.5 to 51.7) *	0.03	2.7 ± 3.2	0.48 (−0.36 to 1.27)
	12min	49.3 ± 2.8 (47.6 to 51.1)	0.08	2.3 ± 3.7	0.41 (−0.40 to 1.22)
SHF	BA	50.7 ± 2.9 (48.8 to 52.5)	——	——	——
	4min	51.7 ± 3.8 (49.2 to 54.1)	0.12	2.0 ± 4.2	0.30 (−0.51 to 1.10)
	8min	52.9 ± 3.4 (50.8 to 55.1) *	0.01	4.5 ± 4.8	0.70 (−0.12 to 1.54)
	12min	50.8 ± 2.1 (49.5 to 52.2)	0.79	0.5 ± 4.9	0.04 (−0.73 to 0.87)

CA: conditioning activity; BA: baseline; CMJ: countermovement jump; SDP: deep squats proximity-to-failure; SDF: deep squats to failure; SHP: half squats proximity-to-failure; SHF: half squats to failure; *: Compared with baseline *p* < 0.05; Δ%: percentage change in CMJ height; *d*: effect size (Cohen’s *d*);

## 4. Discussion

This study aimed to investigate the acute effects of sub-maximal load back squat CAs on CMJ height, with a particular focus on the influence of ROM and intra-set fatigue. The results of this study not only confirmed our original hypothesis but also provided additional insight into the changes in CA mean velocity during the intervention and the temporal characteristics of peak performance. The main findings were as follows: (1) Final repetition mean velocity was significantly lower during deep or half squats performed with repetitions-to-failure protocols compared with proximity-to-failure protocols, with greater velocity loss observed under failure conditions; (2) CMJ height significantly increased after 4- and 8-minute ICRPs across all conditions; (3) The enhancing effects following proximity-to-failure and repetitions-to-failure protocols were comparable; however, the conditions with fewer repetitions led to an earlier peak in CMJ height; (4) For the same number of repetitions, half squats elicited greater improvements in CMJ height compared to deep squats. These findings suggest that coaches and researchers should carefully consider the interplay between ROM and repetition schemes when designing CAs or complex-contrast training programs.

This study found that both the number of repetitions and velocity loss in the repetitions-to-failure protocols were significantly higher than those in the proximity-to-failure protocols (Table 3). This result is consistent with the findings of Chen et al. [55], who studied squat repetitions at 87% 1RM and reported that velocity loss was significantly greater when performing 5 repetitions compared to 3 repetitions. Regarding the timing of the enhancement effect, most studies suggest that the effect can be observed within 3–11 minutes post-CA [25,42,56]. Xu et al. [25] used nonlinear meta-regression analyses to identify an optimal ICRP of 4.5–6.3 min for maximizing jump performance, although this interval may vary according to CA characteristics such as exercise type, loading intensity, and training volume. In the present study, peak CMJ height was observed 4 min post-CA under the proximity-to-failure protocols, whereas peak performance in the repetitions-to-failure protocols was delayed until 8 min post-CA (Fig 3). The peak response observed under the proximity-to-failure protocols was broadly consistent with the optimal ICRP by Xu et al., particularly given that the present study employed a heavy squat load (87% 1RM), which closely approximates the loading conditions associated with the greatest PAPE responses in their analysis. However, unlike the meta-analysis of Xu et al., the present study compared protocols performed with the same exercise modality and relative load while manipulating the level of fatigue induced by the CA. The longer ICRP required under the repetitions-to-failure protocols may be explained by the greater number of repetitions performed and the associated velocity loss, which may have resulted in greater fatigue accumulation and delayed the point at which the enhancement effect outweighed the residual fatigue [57–59]. Therefore, the present findings further support the notion that, even when CA type and loading intensity are held constant, greater intra-set fatigue can shift the optimal ICRP beyond the commonly reported 4.5–6.3 min range. While Sirieiro et al. [35] demonstrated substantial inter-individual variability in the timing of peak responses following repetitions to failure, Kilduff et al. [21] reported enhanced jump performance 8 min after heavy squats. Although the optimal ICRP observed in the present study was consistent with that reported by Kilduff et al. [21] in professional athletes, the underlying mechanisms may not be the same. Specifically, because the non-failure protocol used by Kilduff et al. likely induced relatively little fatigue, the timing of peak performance may have been determined primarily by the time course of enhancement effect. In contrast, the repetition-to-failure protocols employed in the present study may have generated greater fatigue, making fatigue dissipation a more important determinant of the delayed peak response.

Furthermore, as previously mentioned, this study observed significant improvements in jump performance across all conditions. The proximity-to-failure protocols elicited improvements in vertical jump performance that were comparable in magnitude to those observed following the repetitions-to-failure protocols, despite requiring fewer repetitions. Although some differences in effect size estimates were evident, the considerable overlap in their 95% confidence intervals suggests substantial inter-individual variability in responsiveness and indicates that many participants may have responded similarly to both strategies. Consequently, the greater fatigue induced by repetitions to failure does not appear to confer additional performance benefits. Therefore, there appears to be limited practical justification for employing a more fatiguing strategy when comparable performance enhancements can be achieved with fewer repetitions. From a practical perspective, these findings suggest that proximity-to-failure strategies may represent a more time-efficient approach for eliciting PAPE (Table 4). This finding supports the comparable enhancement effects between the 3-repetition proximity-to-failure protocols and the repetitions-to-failure protocols at an intensity of 87% 1RM. Previous studies have shown that repetitions-to-failure protocols can effectively induce enhancement effects [32,33,35]; however, when compared with lower-repetition conditions such as 3 repetitions [31], the magnitude of the effect has varied across studies. These discrepancies may, at least in part, be attributed to differences in ROM between studies. By directly comparing different repetition schemes under matched movement amplitudes, the present study minimized this potential source of variation and allowed the influence of intra-set fatigue to be examined more clearly. The potential role of ROM itself is discussed separately later.

It is noteworthy that the present study observed enhancement following both proximity-to-failure and repetitions-to-failure (7–9 repetitions) protocols. Chen et al. [55] similarly reported improvements in CMJ performance following 3-, 4-, and 5-repetition protocols performed at 87% 1RM despite greater velocity loss under the 5-repetition protocol. However, unlike the present findings, they found no apparent differences in the timing of peak PAPE responses. Given the similarities in load intensity, participant sex, and strength levels between studies, these contrasting findings suggest that the influence of intra-set fatigue may extend beyond the magnitude of performance enhancement and also affect the temporal characteristics of PAPE expression. Therefore, intra-set fatigue may exert a greater influence on the timing of peak performance than on the magnitude of the response itself. Further research is required to determine whether a threshold of fatigue accumulation exists beyond which additional repetitions predominantly alter the temporal profile, rather than the magnitude, of the PAPE response.

As discussed above, both proximity-to-failure and repetitions-to-failure protocols effectively enhanced CMJ height. Beyond this, the present study further demonstrated that half squats were more effective than deep squats in improving peak jump performance (Fig 4). Differences in ROM may partly explain these findings. Compared with quarter squats (knee joint angle ~135°), Esformes and Bampouras [1] suggested that parallel squats performed through a greater range of motion elicited greater activation of the gluteus maximus and associated musculature, thereby facilitating the transition from the eccentric to the concentric phase and enhancing subsequent CMJ performance. Similarly, Kozlenia and Domaradzki [60] demonstrated that isometric squats performed at deeper knee joint angles significantly improved CMJ height. Collectively, these findings indicate that ROM influences the magnitude of PAPE responses, although the relationship does not appear to follow a simple “more is better” pattern. In support of this notion, Krzysztofik et al. [18] reported that a standard bench press produced greater performance enhancement than both larger-ROM and smaller-ROM variations. The authors suggested that the magnitude of PAPE may depend more on the similarity between the CA and the subsequent explosive task than on ROM per se. Accordingly, the effectiveness of a given ROM may be task-specific rather than determined solely by movement amplitude. Importantly, previous studies demonstrating favorable responses to deeper squat positions primarily compared deep or parallel squats with substantially smaller ROM conditions and did not directly contrast deep and half squats [1,60]. Therefore, the present findings do not necessarily contradict earlier studies but rather suggest that the optimal ROM for maximizing PAPE may depend on the characteristics of the subsequent task. Numerous studies have shown that when the movement pattern and ROM of the CA closely resemble those of the subsequent explosive task, the enhancement effect is more pronounced [5,61]. In the present study, participants naturally adopted a squat depth during CMJ testing that more closely resembled the half squat position. Therefore, the greater enhancement effect observed following the half squat may be attributed, at least in part, to the greater biomechanical similarity between the CA and the subsequent jumping task.

This task-specific advantage may be further explained by differences in neuromuscular activation patterns. Gorsuch et al. [62] reported greater activation of the rectus femoris and erector spinae during half squats. Likewise, Vargas-Molina et al. [63] demonstrated that both isometric and dynamic half squat protocols effectively improved CMJ height. Moreover, Silva et al. [64] showed that, although overall quadriceps activation was similar between deep and half squats under equivalent loading conditions, half squats elicited greater activation of the gluteus maximus, biceps femoris, and soleus. Given that these muscles contribute substantially to force production during the propulsion phase of the CMJ, such activation patterns may provide a more favorable neuromuscular stimulus for subsequent explosive performance. Therefore, the superior PAPE response observed following half squats may reflect the combined influence of biomechanical similarity and task-specific neuromuscular activation, potentially together with a lower fatigue cost compared with deep squats, rather than ROM alone. Taken together, these findings suggest that maximizing movement depth is not necessarily required to optimize PAPE. Instead, selecting CAs whose ROM and neuromuscular demands closely resemble those of the subsequent explosive task may be more important for maximizing performance enhancement. Nevertheless, the optimal conditioning strategy is unlikely to be universal and may vary according to individual characteristics, including strength level, training experience, recovery profile, and sport-specific demands. Such factors may partly explain the discrepancies among studies and should be considered when applying PAPE protocols in practice.

A key strength of this study lies in its independent manipulation of ROM and repetition protocols, which, for the first time, clearly elucidates their interactive contribution to the enhancement effect. This provides direct theoretical support for designing more refined warm-up and preparatory strategies. In addition, the use of a linear position transducer enabled objective quantification of movement velocity, providing a reliable and reproducible criterion for monitoring velocity loss and identifying repetitions to failure. However, several limitations should also be acknowledged. Although we sought to minimize confounding influences through a repeated-measures design and standardized procedures, unmeasured variables such as acute fluctuations in recovery capacity, sleep quality, and daily stress may still have contributed to unexplained variability in performance outcomes. Consequently, the magnitude and timing of PAPE responses observed in the present study may not be universally applicable and could vary according to individual characteristics and recovery profiles. These factors represent inherent challenges in studies examining acute neuromuscular performance. Additionally, this study did not include electromyographic assessments of the relevant lower-limb musculature. While muscle activation patterns were discussed, these interpretations were inferred from prior literature rather than directly observed. Future research should consider employing larger sample sizes to better quantify inter-individual variability, as well as incorporating daily status monitoring (e.g., sleep tracking or biomarkers of stress) to account for transient physiological fluctuations. Furthermore, integrating metabolic fatigue indicators, such as blood lactate concentration, alongside repeated assessments of jump performance may provide additional insight into the interaction between fatigue and enhancement effect. Moreover, implementing electromyographic monitoring during the conditioning activities would provide deeper insight into muscle activation strategies and help clarify the mechanisms underlying differential enhancement effect responses. A further limitation of this study is that adherence to the restrictions prior to testing regarding strenuous physical activity, caffeine and alcohol consumption, as well as participants’ sleep patterns and nutritional intake, relied solely on self-reported information and was not objectively monitored or standardized. Consequently, the potential influence of uncontrolled behaviors prior to testing on neuromuscular performance, PAPE responses, and CMJ outcomes cannot be completely excluded. Future studies should consider implementing objective monitoring and standardized pre-testing procedures to further improve experimental control.

## 5. Conclusion

The present study demonstrated that both proximity-to-failure and repetitions-to-failure protocols performed at 87% 1RM effectively enhanced subsequent CMJ performance, with similar enhancement effects observed when ROM was matched. Peak CMJ performance occurred earlier following the proximity-to-failure protocols (4 min post-CA) than following the repetitions-to-failure protocols (8 min post-CA), whereas half squats elicited greater enhancement effects than deep squats. From a practical perspective, these findings suggest that coaches and practitioners may consider using 3 repetitions of the half squat at 87% 1RM as a time-efficient PAPE strategy when explosive performance is required shortly before competition or training. Although repetitions-to-failure protocols produced comparable acute improvements in jump performance, they required a longer recovery period before peak performance was achieved. Therefore, the choice of conditioning strategy should be guided by the time available before performance and the specific demands of the sporting context. As the present study included only resistance-trained male athletes, the applicability of these findings to other populations remains to be established.

## Supporting information

S1 Data Data. (PDF)
