# Supplementary material for: The post-activation performance enhancement of sub-maximal load back squat with varied range of motion and intra-set fatigue on jump height in trained males
Source: PLoS One. 2026 Aug 19;21(8):e0356542. doi: 10.1371/journal.pone.0356542 (PMC13489434; doi:10.1371/journal.pone.0356542)
Supplement: Supplementary file 1 [file pone.0356542.s001.pdf]

Condition	Pro-CA	4min	8min	12min	Initial velocity	Initial velocity	Repetitions
SDP	0.52	0.57	0.53	0.53	0.52	0.51	3.00
SDP	0.46	0.50	0.46	0.46	0.52	0.53	3.00
SDP	0.43	0.48	0.45	0.45	0.54	0.55	3.00
SDP	0.46	0.47	0.45	0.45	0.68	0.47	3.00
SDP	0.47	0.48	0.46	0.45	0.60	0.59	3.00
SDP	0.53	0.56	0.55	0.53	0.50	0.53	3.00
SDP	0.46	0.50	0.50	0.48	0.49	0.51	3.00
SDP	0.46	0.44	0.46	0.43	0.36	0.35	3.00
SDP	0.43	0.44	0.42	0.40	0.56	0.59	3.00
SDP	0.47	0.47	0.46	0.44	0.50	0.44	3.00
SDP	0.45	0.41	0.41	0.41	0.56	0.54	3.00
SDP	0.48	0.51	0.49	0.48	0.58	0.57	3.00
SDF	0.56	0.57	0.60	0.57	0.75	0.47	9.00
SDF	0.46	0.43	0.46	0.43	0.55	0.34	5.00
SDF	0.46	0.46	0.46	0.45	0.69	0.50	12.00
SDF	0.53	0.52	0.53	0.54	0.66	0.55	8.00
SDF	0.52	0.54	0.52	0.53	0.30	0.31	3.00
SDF	0.48	0.51	0.52	0.49	0.50	0.45	5.00
SDF	0.44	0.48	0.49	0.45	0.52	0.33	6.00
SDF	0.50	0.52	0.51	0.52	0.49	0.38	9.00
SDF	0.48	0.48	0.45	0.45	0.50	0.53	6.00
SDF	0.51	0.52	0.54	0.54	0.59	0.40	6.00
SDF	0.50	0.52	0.51	0.54	0.68	0.44	8.00
SDF	0.49	0.51	0.50	0.52	0.63	0.39	7.00
SHP	0.48	0.49	0.50	0.51	0.60	0.57	3.00
SHP	0.47	0.52	0.50	0.49	0.59	0.61	3.00
SHP	0.48	0.51	0.49	0.47	0.69	0.58	3.00
SHP	0.45	0.46	0.46	0.47	0.52	0.41	3.00
SHP	0.47	0.49	0.45	0.49	0.48	0.47	3.00
SHP	0.47	0.47	0.48	0.50	0.56	0.53	3.00
SHP	0.47	0.51	0.46	0.44	0.54	0.42	3.00
SHP	0.46	0.47	0.49	0.48	0.72	0.68	3.00

SHP	0.54	0.53	0.57	0.55	0.64	0.62	3.00
SHP	0.50	0.50	0.52	0.51	0.54	0.35	3.00
SHP	0.49	0.49	0.50	0.49	0.66	0.40	3.00
SHP	0.51	0.52	0.53	0.52	0.63	0.58	3.00

SHF	0.50	0.48	0.49	0.50	0.54	0.35	11.00
SHF	0.50	0.50	0.51	0.49	0.66	0.40	13.00
SHF	0.51	0.48	0.51	0.48	0.59	0.37	10.00
SHF	0.57	0.61	0.59	0.54	0.65	0.50	9.00
SHF	0.47	0.50	0.51	0.53	0.48	0.40	11.00
SHF	0.50	0.51	0.50	0.50	0.49	0.39	5.00
SHF	0.55	0.56	0.56	0.53	0.55	0.36	7.00
SHF	0.51	0.53	0.56	0.52	0.51	0.42	8.00
SHF	0.49	0.51	0.54	0.50	0.53	0.38	12.00
SHF	0.47	0.50	0.52	0.48	0.57	0.43	6.00
SHF	0.52	0.54	0.57	0.53	0.60	0.45	10.00
SHF	0.49	0.48	0.49	0.50	0.62	0.47	9.00
